# Quantitative assessment and Kirschner-wire fixation of an isolated sustentaculum tali fracture in a 7-year-old girl—a case report

**DOI:** 10.3389/fped.2025.1632820

**Published:** 2025-08-21

**Authors:** Fengyong Mao, Lei Ni, Li Ju

**Affiliations:** Department of Pediatric Orthopedics, Children's Hospital of Nanjing Medical University, Nanjing, China

**Keywords:** sustentaculum tali fracture, pediatric, Kirschner wire, case report, quantitative

## Abstract

**Background:**

Isolated sustentaculum tali fractures among pediatric cohorts represent an exceedingly uncommon entity (<1% of all calcaneal fractures), with limited published evidence regarding operative intervention in prepubescent patients. Diagnostic complexities emerge from radiographically indiscernible fracture patterns, mandating cross-sectional imaging modalities. This case study documents the youngest reported patient (7-year-old female) and introduces the first comprehensive morphometric analysis of fracture characteristics and clinical outcomes following surgical management via open reduction and internal fixation (ORIF) utilizing Kirschner wire (K-wire) stabilization.

**Case presentation:**

A 7-year-old female patient presented with right foot pain following a jumping trauma. Clinical assessment demonstrated point tenderness inferior to the medial malleolus with limited subtalar joint mobility. While plain radiography was non-diagnostic, computed tomography (CT) revealed a displaced sustentaculum tali fracture with a fragment measuring 13.62 × 7.89 mm and significant articular angulation (9° in the coronal plane, 16° in the sagittal plane). Surgical management consisted of ORIF utilizing two 1.5 mm K-wires to achieve anatomic reduction. The postoperative protocol included 6 weeks of cast immobilization followed by a structured rehabilitation program. At 12-month follow-up, CT imaging demonstrated complete osseous union with restoration of articular congruity. The patient exhibited optimal functional outcomes with a maximum American Orthopaedic Foot and Ankle Society (AOFAS) hindfoot score of 100, full restoration of ankle range of motion, and complete return to age-appropriate activities without sequelae.

**Conclusions:**

This case underscores the indispensability of CT in the identification of occult sustentaculum tali fractures in pediatric patients. The quantitative parameters observed herein indicate the necessity for patient-specific evaluation protocols. K-wire fixation presents distinct advantages in the pediatric population, notably minimally invasive surgical approach and subsequent facile hardware extraction. Precise anatomical reduction remains paramount for the preservation of hindfoot biomechanics in skeletally immature patients. Although this investigation offers valuable technical insights, multi-institutional prospective studies are warranted to establish definitive surgical criteria and standardized management algorithms.

## Introduction

Sustentaculum tali fractures represent an uncommon injury pattern, with isolated variants constituting <1% of all calcaneal fractures (1). The pediatric incidence is exceptionally rare due to the unique biomechanical properties of the immature skeleton (2), with minimal cases documented in the literature (3). Diagnostic challenges persist as conventional radiography frequently fails to visualize these fractures due to anatomical superimposition, necessitating cross-sectional imaging modalities such as computed tomography (CT) for definitive identification (4, 5).

Evidence-based management protocols for isolated sustentaculum tali fractures in pediatric patients remain poorly defined, particularly regarding surgical indications and fixation methodology. The existing literature predominantly addresses adult populations or adolescents, with a notable paucity of data regarding younger children and quantitative fracture characterization, including fragment morphology and articular involvement (2, 6).

This case report documents the youngest reported patient (7 years) with an isolated sustentaculum tali fracture who underwent successful open reduction and internal fixation (ORIF) utilizing Kirschner wire (K-wire) stabilization. We present the diagnostic algorithm and surgical approach employed, while providing novel quantitative analysis of fragment dimensions, articular surface disruption, and anatomical restoration specific to the pediatric context. These findings may serve as a valuable reference for clinicians managing this rare pediatric orthopedic injury.

## Case presentation

A 7-year-old female of Han ethnicity presented to our emergency department in April 2024 following playground trauma to the right foot after descending from an elevated platform. Clinical examination revealed pedal edema, exquisite tenderness inferior to the medial malleolus, and restricted talocrural motion.

**Figure 1:**
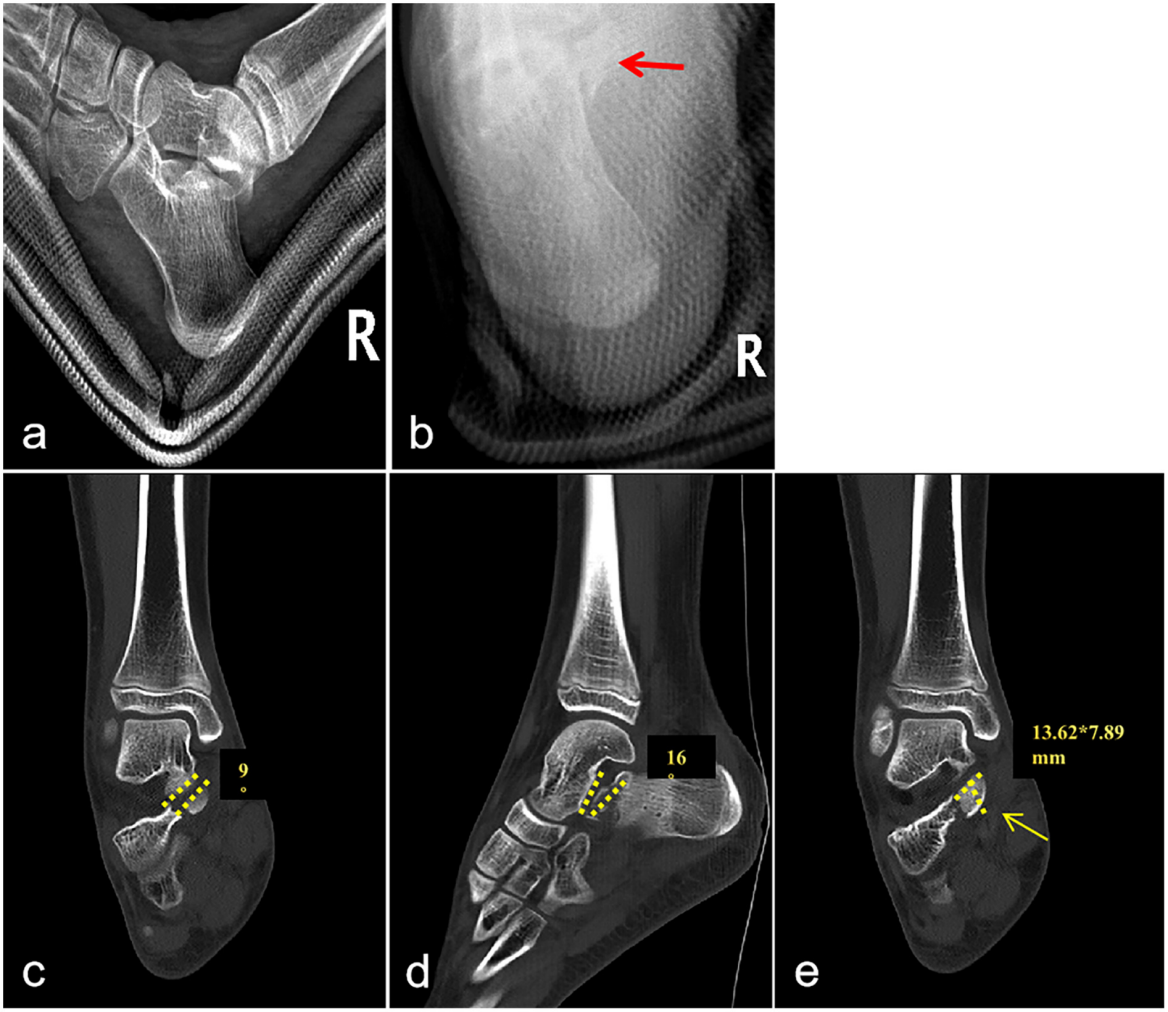
Preoperative x-ray and CT images. **(a)** Lateral view of the calcaneus reveals no clear fracture line. **(b)** Axial view demonstrates a distinct fracture line (red arrow), indicative of a sustentaculum tali fracture, though displacement remains uncertain. **(c)** Coronal CT image demonstrates articular surface disruption with approximately 9° of angulation. **(d)** Sagittal CT image reveals an articular surface angulation of approximately 16°. **(e)** The fracture fragment measures approximately 13.62 mm × 7.89 mm.

Initial radiographic assessment demonstrated no discernible fracture line on lateral projection; however, axial views suggested a sustentaculum tali fracture with questionable displacement. Advanced imaging via CT with three-dimensional reconstruction confirmed a sustentaculum tali fracture fragment (dimensions: 13.62 mm × 7.89 mm). Coronal CT reconstruction demonstrated depression of the talocalcaneal articular surface with 9° angulation, while sagittal reconstruction revealed 16° articular surface angulation (Figure 1).

**Figure 2:**
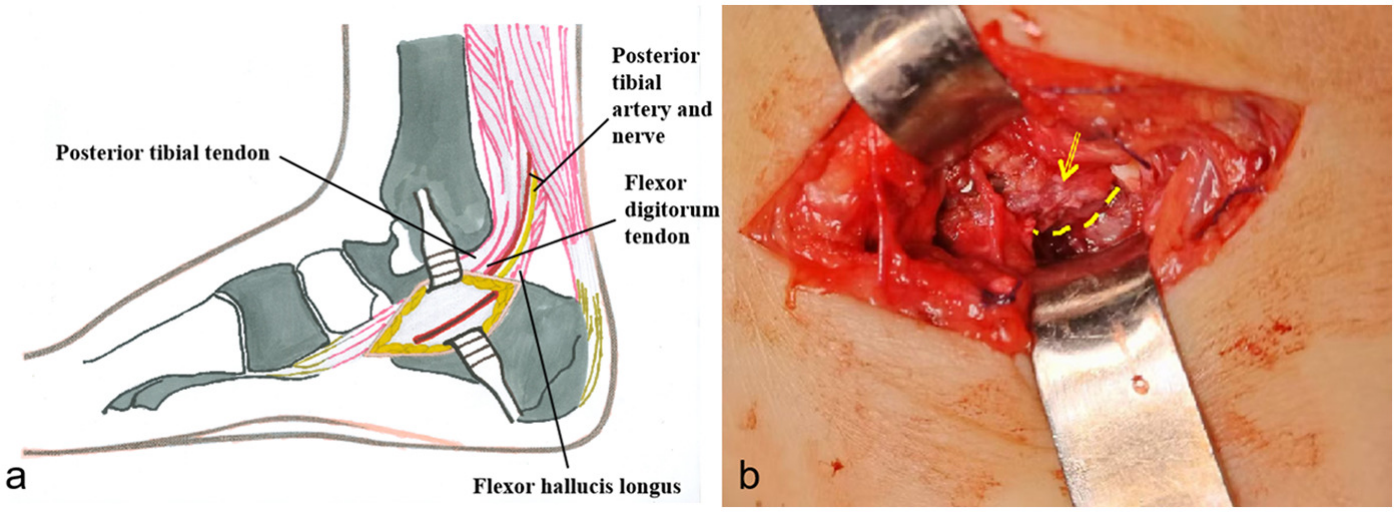
Surgical procedure illustration. **(a)** Surgical approach schematic diagram. **(b)** The arrow indicates the displaced and rotated sustentaculum tali. The dotted line indicates the fracture line.

The irregularity articular surface necessitated surgical intervention to restore joint congruity and mitigate potential sequelae. Following parental consent, the patient underwent ORIF under general anesthesia in supine position with proximal thigh tourniquet control. A 3 cm medial longitudinal incision was created 2 cm distal to the medial malleolus, extending toward the navicular tuberosity (Figure 2). The neurovascular structures, including the posterior tibial tendon and flexor digitorum longus, were identified and protected. After anatomic reduction was achieved, fixation was accomplished using two crossed 1.5 mm K-wires inserted medially with lateral skin exit points.

**Figure 3:**
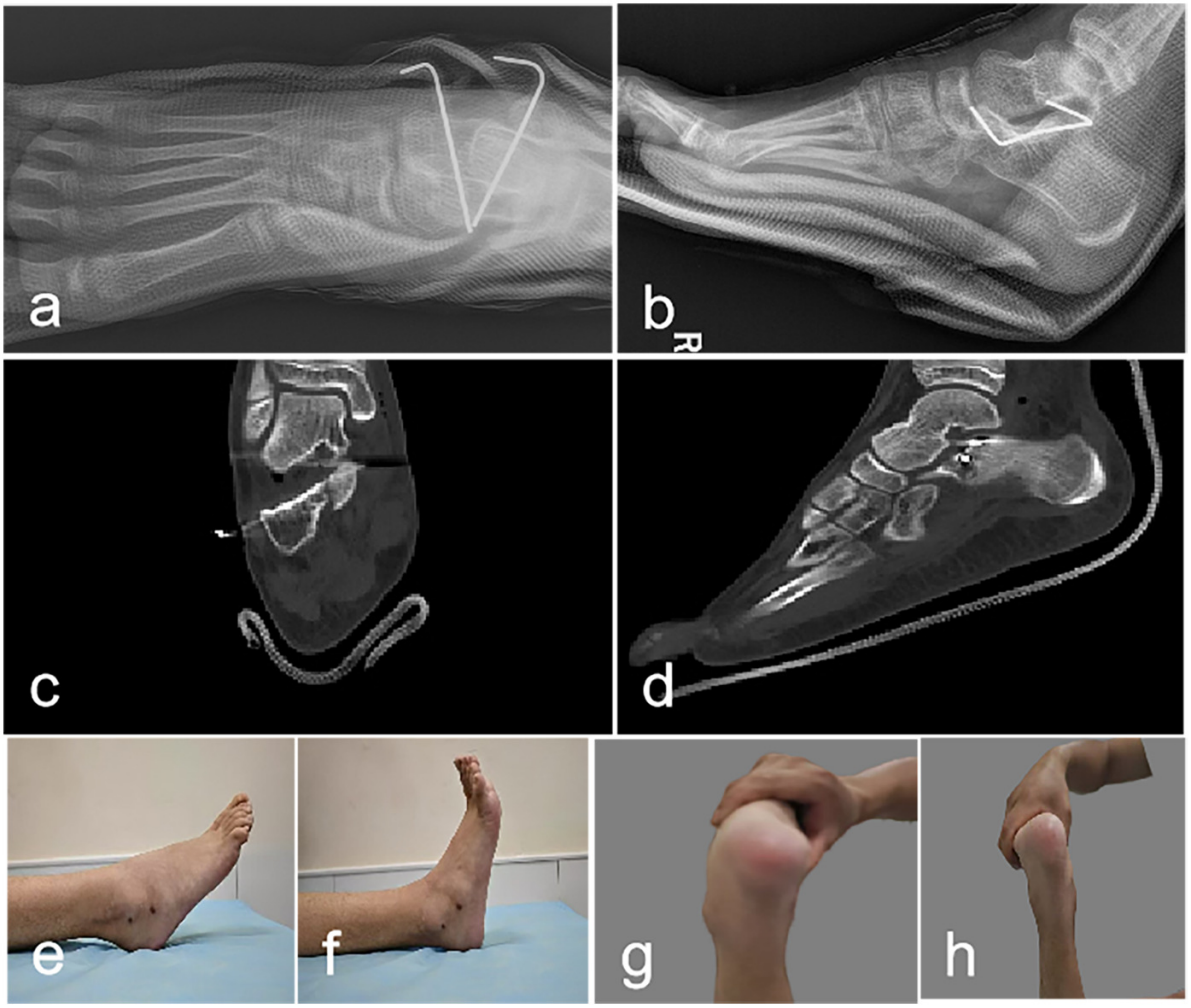
Postoperative x-ray, CT images, and ankle joint range of motion. **(a,b)**: Postoperative radiographic imaging demonstrates adequate fracture reduction with acceptable anatomical alignment; internal fixation hardware maintains stable positioning. **(c,d)** Coronal and Sagittal CT image demonstrates smooth articular surfaces. **(e–h)**: Three-month follow-up examination revealed normal plantarflexion, inversion, and eversion with mild dorsiflexion limitation.

Postoperative management included non-weight-bearing immobilization in a below-knee cast. Radiographic evaluation at 2 weeks demonstrated maintained reduction. At 6 weeks, callus formation was evident radiographically, prompting cast and hardware removal. Progressive weight-bearing and rehabilitation protocols were initiated with excellent patient compliance. Postoperative x-ray, CT images shows a smooth articular surface. Postoperative CT scans in coronal and sagittal planes demonstrate corrected rotational displacement of the sustentaculum tali with a smooth subtalar joint articular surface. Three-month follow-up examination revealed normal plantarflexion, inversion, and eversion with mild dorsiflexion limitation (Figure 3).

One-year post-injury CT imaging demonstrated osseous union with complete resolution of articular incongruity in both coronal and sagittal planes (Supplementary Figure S1). The patient achieved an optimal American Orthopaedic Foot & Ankle Society hindfoot score of 100, with full restoration of ankle kinematics and unrestricted participation in age-appropriate activities without symptomatology.

## Discussion

Sustentaculum tali fractures occurring in isolation are exceedingly uncommon in the pediatric population, with minimal documentation in the literature (2). The inherent elasticity and distinctive biomechanical properties of children's skeletal structures likely account for the rarity of these injuries. Prior to this case, the youngest reported patient with this fracture was 9 years of age (7); therefore, our 7-year-old subject represents the youngest documented case in medical literature. Furthermore, this investigation provides unprecedented quantitative analysis of fracture fragment dimensions and articular surface involvement in pediatric sustentaculum tali fractures, contributing valuable metrics to our understanding of this rare orthopedic injury.

The sustentaculum tali serves as a critical anatomical buttress providing post-surgical stability and maintaining talar alignment following fracture. While intra-articular step-off exceeding 2 mm traditionally necessitates ORIF, articular congruity depends on both translational displacement and rotational alignment. Gitajn and colleagues expanded surgical indications to encompass talocalcaneal articular angulation exceeding 5° in either sagittal or coronal planes, with such incongruity classified as subluxation. Their cohort analysis, demonstrating 20.3% subluxation prevalence, confirms that angular incongruity independently compromises joint stability without necessitating significant translational displacement (8). Notably, sustentacular fractures with concurrent subluxation compromise the biomechanical integrity of reduction, potentially rendering conventional extended lateral approaches—which rely on medial column stability—suboptimal when this foundational support is compromised. Consequently, preoperative CT demonstrating angular deformity >5° warrants comprehensive sustentacular evaluation. Current literature supports medial or combined surgical approaches to facilitate anatomic reduction and stable fixation, thereby restoring calcaneal biomechanical function. In our pediatric case, advanced imaging revealed articular depression with 9° coronal and 16° sagittal angulation, with a displaced fragment measuring 13.62 × 7.89 mm in an isolated sustentacular fracture; a single medial approach with ORIF yielded excellent functional outcomes. As a single case report, these parameters require prospective multicenter validation to establish their applicability to pediatric calcaneal fractures—a critical direction for future investigation.

At present, no consensus protocol exists for the management of sustentaculum tali fractures in the pediatric population. Conservative approaches may suffice for non-displaced or minimally displaced fractures, given children's superior osteogenic capacity. However, fractures with substantial displacement affecting the subtalar articular surface generally necessitate operative management to maintain joint congruence and mitigate potential complications including post-traumatic arthrosis, restricted hindfoot mobility, and functional deficits (7). In the presented case, quantitative assessment via CT revealed significant articular angulation (16° and 9°), providing objective justification for surgical intervention.

Concerning osteosynthesis techniques, Al-Ashhab et al. advocated screw fixation for displaced sustentaculum tali fractures in the adult population (6); however, K-wires were utilized in our investigation for multiple clinical considerations. Primarily, given the minimal fragment dimensions (13.62 mm × 7.89 mm), conventional screws potentially risked iatrogenic comminution due to their greater diameter. Additionally, the trajectory for 1.5 mm smooth K-wire insertion facilitates precise anatomical reduction without articular surface violation, whereas screw placement presents greater technical complexity and typically necessitates serial fluoroscopic confirmation. Furthermore, K-wire extraction can be performed in an ambulatory setting under local anesthesia, markedly diminishing patient morbidity, while screw removal requires subsequent operative intervention. Although percutaneous K-wire fixation carries theoretical risks of pin-site infection, meticulous pin-tract management prevented infectious complications in this case.

Huri and colleagues documented fragment excision with ligamentous reconstruction in a 9-year-old subject (7); however, considering our patient's younger chronological age, we elected to preserve and anatomically reduce the osseous fragment. The sustentaculum tali represents a critical load-bearing structure of the medial subtalar articulation, essential for axial force transmission and maintenance of the medial longitudinal arch architecture, while simultaneously serving as the attachment site for crucial ligamentous and tendinous structures that maintain hindfoot stability and biomechanical function (9). Given the ongoing skeletal maturation in pediatric patients, the long-term developmental sequelae of sustentaculum tali excision remain inadequately characterized. Consequently, we propose that preservation and anatomical reduction should constitute the fundamental treatment algorithm for pediatric sustentaculum tali fractures, with K-wire or suture fixation prioritized even for diminutive or comminuted fragments. The patient's 12-month postoperative American Orthopaedic Foot and Ankle Society score of 100 points and radiographic confirmation of articular congruity via CT substantiate the clinical efficacy and safety profile of ORIF utilizing K-wire in the pediatric population.

The findings of this investigation indicate that healthcare providers should exercise heightened clinical vigilance for sustentaculum tali fractures among pediatric populations presenting with height-related fall trauma, localized tenderness inferior to the medial malleolus, and radiographically indeterminate findings. Expedited CT is advised for diagnostic confirmation. Surgical intervention warrants consideration when quantitative CT analysis demonstrates significant articular involvement. K-wire fixation represents the preferred stabilization method due to its efficacy with small osseous fragments and subsequent removal simplicity.

This investigation is constrained by its case report methodology, limiting the generalizability of its conclusions. Subsequent research initiatives should focus on expanding the clinical database and extending follow-up duration to elucidate optimal management protocols, comparative efficacy of fixation techniques, and long-term sequelae associated with sustentaculum tali fractures in the pediatric population.

## Conclusion

This case report documents the youngest patient (7 years old) with an isolated sustentaculum tali fracture and provides unprecedented quantitative analysis of fracture fragment dimensions and articular surface disruption in the pediatric population. The findings underscore the necessity of surgical intervention for displaced sustentaculum tali fractures in children, particularly with articular involvement, to restore anatomical alignment and joint congruity. Following ORIF with K-wires, excellent functional outcomes were achieved, demonstrating the procedure's safety profile and clinical efficacy in the pediatric cohort. This report contributes significant clinical evidence and quantitative reference parameters to the literature, potentially guiding orthopedic surgeons in the diagnosis and management of this rare pediatric tarsal fracture, thereby enhancing evidence-based clinical decision-making.

## Data Availability

The datasets presented in this study can be found in online repositories. The names of the repository/repositories and accession number(s) can be found in the article/Supplementary Material.

## Supplementary material

Supplementary Figure S1 Follow-up CT images obtained at 1 year postoperatively. Radiological evaluation confirms complete fracture union. CT images demonstrate anatomical restoration of the articular surface. Coronal (a) and sagittal (b) CT views confirm restoration of normal articular surface alignment.
