# Supplementary figures and images for: Quantitative assessment and Kirschner-wire fixation of an isolated sustentaculum tali fracture in a 7-year-old girl—a case report
Source: Front Pediatr. 2025 Aug 21;13:1632820. doi: 10.3389/fped.2025.1632820 (PMC12408644; doi:10.3389/fped.2025.1632820)

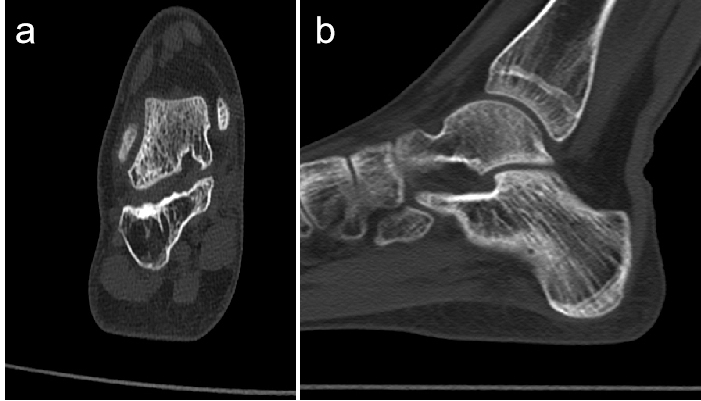

Supplement: Supplementary Figure S1 — Follow-up CT images obtained at 1 year postoperatively. Radiological evaluation confirms complete fracture union. CT images demonstrate anatomical restoration of the articular surface. Coronal (a) and sagittal (b) CT views confirm restoration of normal articular surface alignment. [file Image1.tif]
